# Trends in tobacco use and tobacco cessation counselling codes among Medicare beneficiaries, 2001–2014

**DOI:** 10.1186/s12913-019-4368-7

**Published:** 2019-08-05

**Authors:** Shawn P. E. Nishi, Jie Zhou, Young-Fang Kuo, Gulshan Sharma, James Goodwin

**Affiliations:** 1 Department of Internal Medicine, University of Texas Medical Branch, 301 University Blvd, Galveston, TX 77555 USA; 2 Department of Preventive Medicine, University of Texas Medical Branch, 301 University Blvd, Galveston, TX 77555 USA; 3 Sealy Center on Aging, University of Texas Medical Branch, 301 University Blvd, Galveston, TX 77555 USA; 4 Division of Pulmonary Critical Care Medicine & Sleep, University of Texas Medical Branch, 301 University Blvd, Galveston, TX 77555-0561 USA

**Keywords:** Medicare, Tobacco cessation, Tobacco counselling, Tobacco use, Tobacco prevalence

## Abstract

**Background:**

Analysis of Medicare data is often used to determine epidemiology, healthcare utilization and effectiveness of disease treatments. We were interested in whether Medicare data could be used to estimate prevalence of tobacco use. Currently, data regarding tobacco use is derived from *Behavioral Risk Factor Surveillance System* (BRFSS) survey data.

We compare administrative claims data for tobacco diagnosis among Medicare beneficiaries to survey (BRFSS) estimates of tobacco use from 2001 to 2014.

**Methods:**

Retrospective cross-sectional study comparing tobacco diagnoses using International Classification of Disease, Ninth Revision (ICD-9) codes for tobacco use in Medicare data to BRFSS data from 2001 to 2014 in adults age ≥ 65 years. Beneficiary data included age, gender, race, socioeconomic status, and comorbidities. Tobacco cessation counselling was also examined using Healthcare Common Procedure Coding System codes.

**Results:**

The prevalence of Medicare enrollees aged ≥65 years who had a diagnosis of current tobacco use increased from 2.01% in 2001 to 4.8% in 2014, while the estimates of current tobacco use from BRFSS decreased somewhat (10.03% in 2001 vs. 8.77% in 2014). However, current tobacco use based on Medicare data remained well below the estimates from BRFSS. Use of tobacco cessation counselling increased over the study period with largest increases after 2010.

**Conclusions:**

The use of tobacco-related diagnosis codes increased from 2001 to 2014 in Medicare but still substantially underestimated the prevalence of tobacco use compared to BRFSS data.

## Background

Tobacco use remains the leading cause of preventable morbidity and mortality in the United States and is a major contributor to the nation’s increasing medical costs [1, 2]. Smoking is a modifiable risk factor and smoking cessation at any age decreases the development of further smoking-related disease and improves life expectancy over that of those who continue to smoke [2, 3]. Comprehensive identification of smokers is pivotal to achieving these goals.

Most tobacco use prevalence data is derived from large surveys conducted via phone [4–6]. An alternative method to estimate the prevalence of various health conditions is to use health care claims data [7]. The use of diagnosis codes in administrative data to identify common diseases has been shown to have variable sensitivity and specificity [8–10]. For example, combinations of *diagnosis* and *procedure* codes for patients with Medicare or commercial health insurance have good sensitivity and specificity in identifying incident cancer diagnoses, compared to a gold standard of tumor registry data [11]. In contrast, the use of administrative data to evaluate *risk factors* for a disease or condition are often less reliable. For instance, tobacco-related diagnosis, such as tobacco use disorder or history of tobacco use, are insensitive in estimating the prevalence of current and past smokers, but have a high specificity [10, 12–14]. In 2000–2001, Kokotailo et al. reported a 7% sensitivity using International Classification of Disease, Ninth Revision (ICD-9) codes for current or past tobacco use and > 98% specificity [12]. This results in grossly underestimated smoking prevalence rates using administrative data. This lack of sensitivity is presumably a major reason that tobacco use is not included in the most common comorbidity measures used in analyses of administrative data [15–17].

Recently, several initiatives, such as Healthy People 2020, focus on three objectives related to smoking: decreasing the proportion of U.S. adults who smoke cigarettes to ≤12.0%, increasing those who attempt to quit smoking cigarettes to ≥80.0% and increasing recent smoking cessation success to ≥8.0% [18]. The Surgeon General’s report and various clinical practice guidelines heavily focus on increasing identification and recommend universal screening for tobacco use among all individuals seen in any healthcare setting [2, 19, 20]. In 2005, the Center for Medicare and Medicaid Services (CMS) introduced reimbursement for tobacco cessation counselling in patients with tobacco-related disease [21]. Reimbursement for activities related to a specific diagnosis may incentivize more complete use of that diagnosis in claims data [22].

Given these recent financial and medical initiatives, we hypothesized that tobacco use diagnoses and tobacco cessation counselling codes have increased and that estimates of tobacco use diagnoses in administrative claims data might better approximate estimates obtained from surveys in the past 10 years. The purpose of this study was to examine trends of tobacco diagnoses and tobacco cessation counselling using national Medicare claims data and compare it with estimates of tobacco use obtained by the Behavioral Risk Factor Surveillance System (BRFSS), a national, population-based survey.

## Methods

### Data sources

This is a retrospective cross-sectional study comparing the trends in the prevalence of tobacco diagnoses and tobacco cessation counselling in Medicare claims data to prevalence of tobacco use estimated by the BRFSS. We used the 5% sample of national Medicare enrollees from January 1, 2001 through December 31, 2014, and BRFSS from January 1, 2001 through December 31, 2014. This study was approved by the University of Texas Medical Branch Institutional Review Board (IRB 17–109) and informed consent was not obtained. Waiver of consent is was granted under the U.S. Federal Policy for the Protection of Human Subjects 45 CFR 46.116 which states that the nature of the study involves no more than minimal risk to subjects, research could not be carried out practicably without the waiver or alteration, and waiver will not adversely affect the rights and welfare of the subjects.

### Medicare

The Centers for Medicare & Medicaid Services, CMS, is part of the Department of Health and Human Services (HHS) which provides hospital and medical insurance for Americans. Over 98% of adults in the United States age ≥ 65 years are enrolled in Medicare, which includes > 55 million beneficiaries. The CMS selects a random sample of 5% of Medicare beneficiaries based on the eighth and ninth digits (05, 20, 45, 70, 95) of their health insurance claim number. The resulting standard data is available for research purposes and has been shown to be representative of the entire cohort [23]. In this study we used the following Medicare data files: Medicare Denominator File for demographic and enrollment information, the Carrier File for physician claims, the Outpatient Statistical Analysis File for outpatient claims and the Medicare Provider Analysis and Review File for inpatient claims. The data that support the findings of this study are available from CMS but restrictions apply to the availability of these data, which were used under license for the current study, and so are not publicly available.

### BRFSS

The BRFSS data is used to estimate the current smoking rate in each state. The BRFSS conducts more than 500,000 health-related telephone surveys annually in persons > 18 years of age in all 50 states as well as the District of Columbia and three U.S. territories. These surveys ask about health-related risk behaviors, chronic health conditions and use of preventive services. Surveys are conducted by each state and use iterative proportional fitting or raking of variables in the weighting process to reduce the potential for selection bias while increasing representativeness of estimates. Variables used include age, race and ethnicity, sex, geographic region within states, education level, marital status, type of phone ownership and home ownership. It is the largest, continuously conducted health survey in the world. The data obtained for the study is publically available at their website [24].

### Study cohort

#### Medicare

We developed cohorts for each year from 2001 through 2014. The 2001 cohort included all beneficiaries aged 65 years or older on January 1st, 2001, with complete Medicare Part A and B coverage and no health maintenance organization (HMO) enrollment in the previous year; 2007 and 2014 cohorts were selected in the same way.

#### BRFSS

To estimate the trends in tobacco use, we constructed yearly cohorts from 2001 to 2014 for BRFSS data (16). In order to compare same aged subjects in both BRFSS and Medicare, we restricted BRFSS surveys to include only those aged 65 years or older in 2001 to be in the 2001 year cohort; and the same cohort selection procedure was used for each year through 2014.

### Variables

Medicare enrollment files were used to categorize subjects by age, gender and race/ethnicity (non-Hispanic white, black, Hispanic, other). Low socioeconomic status was identified by Medicaid eligibility status “yes”. Chronic Obstructive Pulmonary Disease (COPD) or Emphysema diagnosis was identified by reviewing all ICD-9 codes 490, 491, 492, 496 associated with inpatient and outpatient billing claims for the previous 12 months. We also calculated a comorbidity score for the remaining comorbidities included in the Elixhauser comorbidity score (48).

### Outcome measure

Our outcomes of interest in the Medicare files were the use of tobacco diagnosis and tobacco cessation counseling codes over the study period. Beneficiaries were considered to have tobacco cessation counseling if they had any one of following Healthcare Common Procedure Coding System (HCPCS) or Current Procedure Terminology (CPT) codes: G0375, G0436 and 99,406 (smoking or tobacco cessation counselling 3–10 min); G0376, GO437 and 99,407 (smoking or tobacco cessation counselling > 10 min). Multiple codes were used to include all updated codes over the research time period for tobacco cessation counselling. Using Medicare diagnoses, we estimated the rate of prior smokers and current smokers for each year. Prior smokers were defined in Medicare data if they had the code V15.82 (history of tobacco use). Current smokers were defined by ICD-9 codes 305.1 (tobacco use disorder) or 989.84 (toxic effect of tobacco). We also estimated the yearly prevalence of smoking in the BRFSS data. BRFSS has a variable named ‘Smoking Status’. It has four levels: 1 (Current smoker – now smokes every day), 2 (Current smoker – now smokes some days), 3 (Former smoker), and 4 (Never smoked). We defined Current smokers as those with a Smoking Status of 1.

### Statistical analysis

In the Medicare data, for each year from 2001 to 2014, we calculated the percentage of current smokers, former smokers and patients who received tobacco cessation counseling. Descriptive analysis was used to summarize the patient characteristics and rate of smokers for the 2001, 2007 and 2014 cohorts. Also, we identified the number of beneficiaries with tobacco diagnosis and tobacco cessation counseling claims to investigate the trend in rates of tobacco use and cessation from 2001 to 2014. The percentage of beneficiaries with tobacco cessation counseling claims (99,406, 99,407, G0436, G0437, G0375, G0376) were calculated for each year from 2001 to 2014. We then analyzed the time trends in prevalence of tobacco diagnoses and tobacco cessation counseling claims using joinpoint analysis with a non-linear model to identify change points and 95% confidence intervals, and also slopes between the change points [25]. Statistical significance for the joinpoint model analysis was at *p* < 0.0001. Self-reported survey rates of former and current tobacco use using BRFSS were compared with rates from the 5% sample national Medicare claims data. A multivariate repeated logistic regression model estimated from Generalized Estimating Equations (GEE) was used to check whether the rate of tobacco diagnostic codes changed significantly between 2001 and 2014 by each beneficiary characteristics. In this GEE model, the interaction between beneficiaries characteristics [age, gender, race/ethnicity, Medicaid eligibility (Yes/No), comorbidity score (0–1, 2–3, 4+), COPD/Emphysema] and year of Medicare claims were included. Since all of these interactions were significant, the adjusted odds ratio on the change in rate of tobacco diagnostic code by characteristics was estimated from this model. SAS version 9.4 (SAS Institute, Cary, NC) was used for all statistical analyses.

## Funding

This research was supported by The Cancer Preventive and Research Institute of Texas (CPRIT, RP1607674) and the National Cancer Institute (K05CA135923). The funding sources had no role in the study conception, design, conduct or analysis. This work has not been and modified by the funding source or approval of the manuscript was not required.

## Results

Characteristics of the cohort in three representative years (2001, 2007, 2014) over the study period, as well as the percent who had any tobacco diagnosis as a current or former smoker in those years are shown in Table 1. In all 3 years, the prevalence of tobacco diagnoses was higher in younger subjects, in males and in those eligible for Medicaid. There were also clear increases in the prevalence of tobacco diagnoses over time in all categories. This increase is shown more clearly in Table 2, with the adjusted odds ratios of tobacco diagnoses prevalence in 2014 v. 2001 stratified by the characteristics of Medicare enrollees. The increases were larger among older enrollees and those with increasing comorbidity.

**Table 1:** Baseline characteristics of Medicare beneficiaries and percentage tobacco use diagnoses^1^ in 2001, 2007 and 2014

	2001			2007			2014		
	N (%) or Mean (Std)	% with tobacco diagnosis	ORs	N (%) or Mean (Std)	% with tobacco diagnosis	ORs	N (%) or Mean (Std)	% with tobacco diagnosis	ORs
All	1,190,827	3.61%		1,234,434	5.49%		1,362,211	12.97%	
Age									
66–69	243,110 (20.42%)	4.62%	Reference	225,138 (18.24%)	7.14%	Reference	326,808 (23.99)	13.01%	Reference
70–74	312,072 (26.21%)	4.39%	0.92 (0.89–0.94)	315,571 (25.56%)	6.54%	0.88 (0.86–0.90)	328,549 (24.12%)	14.22%	1.01 (0.99–1.02)
75–79	276,067 (23.18%)	3.68%	0.74 (0.71–0.76)	278,436 (22.56%)	5.67%	0.72 (0.70–0.74)	259,600 (19.06%)	13.93%	0.91 (0.90–0.93)
80–84	190,697 (16.01%)	2.78%	0.54 (0.52–0.56)	217,819 (17.65%)	4.51%	0.55 (0.54–0.57)	205,506 (15.09%)	13.15%	0.81 (0.80–0.83)
85+	168,881 (14.18%)	1.52%	0.30 (0.28–0.31)	197,470 (16.00%)	2.74%	0.33 (0.32–0.34)	241,748 (17.75%)	10.01%	0.58 (0.57–0.60)
Gender									
Female	715,118 (60.05%)	2.87%	Reference	724,263 (58.67%)	4.42%	Reference	815,915 (59.90%)	10.70%	Reference
Male	475,709 (39.95%)	4.71%	1.54 (1.51–1.57)	510,171 (41.33%)	7.00%	1.52 (1.50–1.55)	546,296 (40.10%)	16.35%	1.66 (1.64–1.68)
Race/Ethnicity									
White	1,054,364 (88.54%)	3.71%	Reference	1,040,394 (84.28%)	5.70%	Reference	1,136,026 (83.40%)	13.42%	Reference
Black	88,485 (7.43%)	3.30%	0.84 (0.81–0.88)	92,454 (7.49%)	5.55%	0.93 (0.90–0.96)	101,084 (7.42%)	13.86%	0.96 (0.94–0.98)
Hispanic	21,744 (1.83%)	1.89%	0.41 (0.37–0.46)	64,186 (5.20%)	3.21%	0.49 (0.46–0.52)	69,191 (5.08%)	8.46%	0.53 (0.52–0.55)
Others	26,234 (2.20%)	1.93%	0.46 (0.42–0.50)	37,400 (3.03%)	3.31%	0.54 (0.50–0.57)	55,910 (4.10%)	7.64%	0.52 (0.50–0.54)
Medicaid eligible									
Yes	151,482 (12.72%)	4.40%	Reference	167,008 (13.53%)	6.51%	Reference	174,648 (12.82%)	16.00%	Reference
No	1,039,345 (87.28%)	3.49%	0.73 (0.71–0.75)	1,067,426 (86.47%)	5.33%	0.78 (0.76–0.80)	1,187,563 (87.18%)	12.52%	0.80 (0.79–0.81)
Elixhauser Comorbidity									
0–1	770,969 (64.74%)	3.36%	Reference	699,085 (56.63%)	4.86%	Reference	691,542 (50.76%)	9.60%	Reference
2–3	296,602 (24.91%)	3.82%	1.06 (1.04–1.09)	354,357 (28.71%)	6.00%	1.16 (1.14–1.19)	412,131 (30.25%)	13.97%	1.44 (1.42–1.46)
4+	123,256 (10.35%)	4.63%	1.07 (1.03–1.10)	180,992 (14.66%)	6.92%	1.09 (1.06–1.120	258,556 (18.98%)	20.37%	1.90 (1.87–1.93)
COPD or Emphysema									
Yes	190,128 (15.97%)	8.85%	Reference	204,062 (16.53%)	13.35%	Reference	216,620 (15.90%)	30.11%	Reference
No	1,000,699 (84.03%)	2.61%	0.28 (0.27–0.29)	1,030,372 (83.47%)	3.93%	0.27 (0.26–0.28)	1,145,591 (84.10%)	9.73%	0.28 (0.28–0.29)

1. Tobacco use: identified using ICD-9 codes 305.1, 989.84, and V15.82

2. Medicaid eligibility status: based on whether the patient was eligible for state buy-in coverage provided by the Medicaid program for at least 1 month or in low-income subsidy part D program during the study year

3. Elixhauser Comorbidity components: congestive heart failure, valvular disease, pulmonary circulation disorders, peripheral vascular disorders, hypertension, paralysis, other neurological disorders, diabetes-uncomplicated, diabetes-complicated, hypothyroidism, renal failure, liver disease, peptic ulcer disease excluding bleeding, AIDS (acquired immune deficiency syndrome), lymphoma, metastatic cancer, solid tumor without metastasis, rheumatoid arthritis/collagen vascular diseases, coagulopathy, obesity, weight loss, fluid and electrolyte disorders, blood loss anemia, deficiency anemia, alcohol abuse, drug abuse, psychoses, and depression;

4. COPD/Emphysema: identified using ICD-9 codes 490, 491, 492, 496

**Table 2:** Adjusted Odds Ratios for Tobacco use^a^ diagnoses in 2001 and 2014 in each patient characteristics’ category

	2001		2014		ORs 2014 vs. 2001
	N (%) or Mean (Std)	% with tobacco diagnoses	N (%) or Mean (Std)	% with tobacco diagnoses	
All	1,190,827	3.61%	1,362,211	12.97%	
Age					
66–69	243,110 (20.42%)	4.62%	326,808 (23.99)	13.01%	4.13 (3.96–4.31)
70–74	312,072 (26.21%)	4.39%	328,549 (24.12%)	14.22%	4.55 (4.37–4.74)
75–79	276,067 (23.18%)	3.68%	259,600 (19.06%)	13.93%	5.11 (4.90–5.33)
80–84	190,697 (16.01%)	2.78%	205,506 (15.09%)	13.15%	6.22 (5.93–6.52)
85+	168,881 (14.18%)	1.52%	241,748 (17.75%)	10.01%	8.08 (7.65–8.53)
Gender					
Female	715,118 (60.05%)	2.87%	815,915 (59.90%)	10.70%	5.24 (5.04–5.44)
Male	475,709 (39.95%)	4.71%	546,296 (40.10%)	16.35%	5.68 (5.46–5.91)
Race/Ethnicity					
White	1,054,364 (88.54%)	3.71%	1,136,026 (83.40%)	13.42%	4.77 (4.68–4.85)
Black	88,485 (7.43%)	3.30%	101,084 (7.42%)	13.86%	5.47 (5.25–5.71)
Hispanic	21,744 (1.83%)	1.89%	69,191 (5.08%)	8.46%	6.24 (5.63–6.91)
Others	26,234 (2.20%)	1.93%	55,910 (4.10%)	7.64%	5.43 (4.94–5.97)
Medicaid eligible^b^					
Yes	151,482 (12.72%)	4.40%	174,648 (12.82%)	16.00%	5.20 (4.99–5.42)
No	1,039,345 (87.28%)	3.49%	1,187,563 (87.18%)	12.52%	5.72 (5.49–5.95)
Elixhauser Comorbidity^c^					
0–1	770,969 (64.74%)	3.36%	691,542 (50.76%)	9.60%	4.07 (3.92–4.24)
2–3	296,602 (24.91%)	3.82%	412,131 (30.25%)	13.97%	5.51 (5.29–5.74)
4+	123,256 (10.35%)	4.63%	258,556 (18.98%)	20.37%	7.22 (6.90–7.56)
COPD or Emphysema^d^					
Yes	190,128 (15.97%)	8.85%	216,620 (15.90%)	30.11%	5.38 (5.18–5.60)
No	1,000,699 (84.03%)	2.61%	1,145,591 (84.10%)	9.73%	5.52 (5.31–5.74)

^a^Tobacco use: identified using ICD-9 codes 305.1, 989.84, and V15.82

^b^Medicaid eligibility status: based on whether the patient was eligible for state buy-in coverage provided by the Medicaid program for at least 1 month or in low-income subsidy part D program during the study year

^c^Elixhauser Comorbidity components: congestive heart failure, valvular disease, pulmonary circulation disorders, peripheral vascular disorders, hypertension, paralysis, other neurological disorders, diabetes-uncomplicated, diabetes-complicated, hypothyroidism, renal failure, liver disease, peptic ulcer disease excluding bleeding, AIDS (acquired immune deficiency syndrome), lymphoma, metastatic cancer, solid tumor without metastasis, rheumatoid arthritis/collagen vascular diseases, coagulopathy, obesity, weight loss, fluid and electrolyte disorders, blood loss anemia, deficiency anemia, alcohol abuse, drug abuse, psychoses, and depression;

^d^COPD/Emphysema: identified using ICD-9 codes 490, 491, 492, 496

**Fig. 1:**
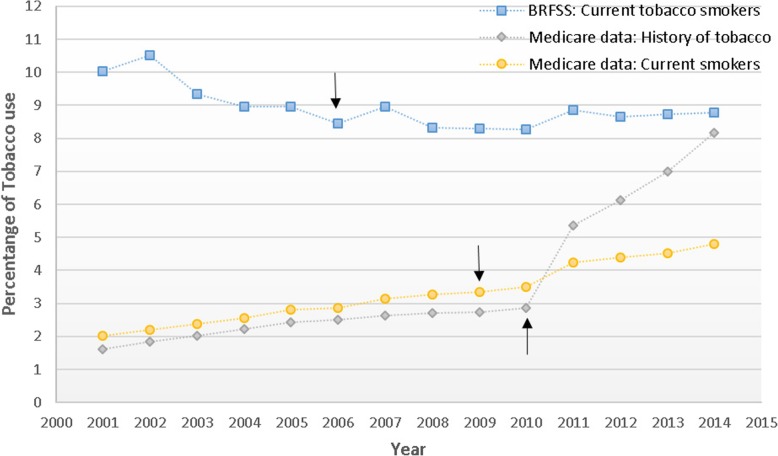
Comparison of the percentage with current and former tobacco diagnosis of Medicare beneficiaries and *Behavioral Risk Factor Surveillance System* (*BRFSS*) data by year from 2001 to 2014 in individuals age 65 and older. Arrows indicate points with significant changes in slope, by joinpoint analysis

Next, we compared the prevalence of diagnostic codes of current and former smokers among Medicare beneficiaries to the survey estimates of the prevalence of current and former smokers from *BRFSS (*Fig. 1*). *In 2001, the prevalence of current smokers estimated by Medicare claims data in those ≥65 was much lower than survey data estimated by BRFSS (2.01% vs 10.03%). The 2001 estimate of current smokers based on Medicare diagnoses was approximately 20% that of BRFSS data. By 2014, the Medicare estimate was approximately 55% of the BRFSS estimate.

Time trend analysis showed significant increase in current and history if tobacco use diagnosis estimated by Medicare data. Current smoker diagnosis increased with a significant change in slope in 2009. Before 2009, the prevalence of current tobacco use diagnosis increased at 0.17% per year. After 2009 the slope increased to 0.29% per year. Diagnosis of history of tobacco use rose from 2001 to 2010, with a slope of 0.18% per year. Thereafter, the slope rose sharply, with a slope of 1.30% per year. The prevalence of former smokers in BRFSS was > 40% and did not vary much over time (data not shown in Figure).

**Fig. 2:**
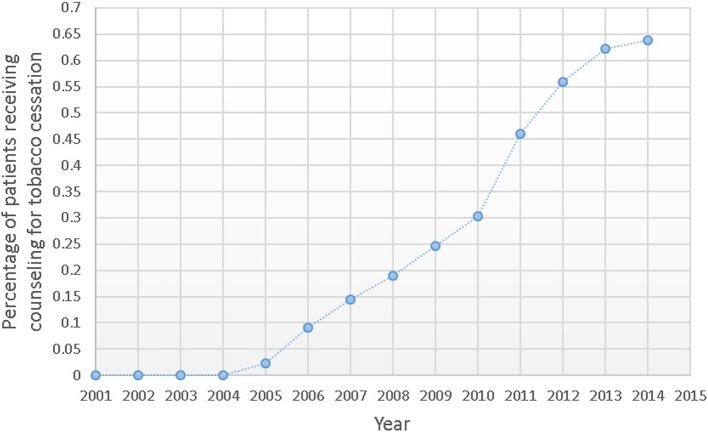
Percentage of Medicare beneficiaries with tobacco cessation counseling claims (99,405, 99,407, G0436, G0437, G0375, G0376) from 2001 to 2014. Before 2005 there were no codes for tobacco cessation counseling

We next explored the time trends in Medicare reimbursement for tobacco cessation counselling in Medicare claims (Fig. 2). CMS reimburses up to 2 individual cessation attempts per year. Each attempt may include up to 4 counselling sessions for a total of up to 8 reimbursable sessions per year [25]. In 2005, Medicare introduced reimbursement for tobacco cessation counselling for *symptomatic* tobacco user, and modified to include *asymptomatic* tobacco users in 2010. Figure 2 presents the percent of Medicare beneficiaries with claims for tobacco cessation counselling over the study period. The percent of beneficiaries with billing codes for tobacco cessation counselling in 2005 was 0.03%. The rate of counseling increased steadily, to 0.35% in 2010. There was a larger increase between 2010 and 2011, coincident with the change in CMS policy to reimburse physicians for counselling asymptomatic tobacco users, with an apparent plateau in rate over 2012–2014 at approximately 0.68% per year. Joinpoint analysis of tobacco cessation claims over time detected no change in slope after the introduction of the codes for tobacco cessation counseling in 2005.

## Discussion

Our study compares administrative claims data for tobacco diagnosis among Medicare beneficiaries to survey (BRFSS) estimates of tobacco use from 2001 to 2014. The prevalence of patients with Medicare tobacco-related diagnosis codes increased over the study period. Although the prevalence of tobacco diagnosis codes in Medicare data more closely approximated BRFSS tobacco use estimates in 2014 than in 2001, Medicare administrative data continue to substantially underestimate tobacco use rates in the elderly.

The reasons for the increase in identification of tobacco use in our study over time, especially after 2010, are likely multifactorial. Multiple improvement efforts include prompts to assist with consistent identification of smokers at every health care encounter [19, 26–28]. Initiatives, such as Healthy People 2000 and 2010 have stressed the importance of recognizing and treating tobacco dependence, as have clinical practice guidelines [18, 20]. In addition, the number of evidence-based treatments for tobacco dependence has increased. The initial report by the National Lung Screening Trial, on the efficacy of low dose CT screening in reducing lung cancer mortality, also emphasized the importance of a healthcare team to provide a “teachable moment” of tobacco cessation benefits [29–31]. Expanded Medicare reimbursement for tobacco cessation counselling to include all patients identified with tobacco abuse occurred in 2010, which also coincided with increased CMS reimbursement [32]. In the same year, the Affordable Care Act mandated coverage of tobacco cessation interventions with no co-pays among private, Medicaid and Medicare beneficiaries [33, 34]. And in 2011, Meaningful Use regulation provided financial incentives to improve health care with information technology to include documentation of smoking status [35]. By 2013, more than 50% of US hospitals participated in Meaningful Use programs [36].

Despite these efforts highlighting and incentivizing medical providers to assist in tobacco use identification for cessation counselling and treatment, overall, Medicare administrative data for diagnosis of tobacco use is half the prevalence shown by BRFSS survey data. A recent similar study using National Inpatient Sample data reported a substantially lower prevalence of tobacco use compared to BRFSS data [37]. Providers may be hesitant to add tobacco use diagnoses due to “tobacco rating” of health insurance plans which increase health care premiums for tobacco users up to 50% [38]. Diagnostic coding is derived from a complex combination of provider identification, documentation, clerical interpretation and assignment of diagnostic codes for tobacco use or treatment, which further leads to under-recognition when reviewing administrative data. This is evidenced in studies using Natural Language Processing or manual chart audit which highlight the large discrepancy between chart notation and administrative codes in the assessment of or treatment for tobacco use [13, 14]. Several randomized controlled trials adding tobacco status as a vital sign to the electronic health record improved on this process [39–41]. Often cited provider reasons for not providing tobacco cessation counselling include difficulty in managing multiple competing disease conditions in a time-constrained appointment with less focus on risk factors, such as tobacco use, than disease. Also, providers may perceive themselves as ineffective counsellors, may lack confidence or knowledge in discussing smoking cessation with their patients or may feel personal discomfort providing counselling [42–44]. Additionally, providers who smoke are shown to be less likely than non-smokers or ex-smokers to direct and counsel their patients to quit [45]. Identification of smokers may also remain low because of patients’ or providers’ nihilistic attitude toward cessation or lack of knowledge of the availability, safety or efficacy of pharmacologic treatments, which are first line therapy in clinical practice guideline recommendations. This is evidenced in a recent study showing no change in varenicline and bupropion prescription since 2007, with only 16% of tobacco users ever having filled a prescription for a smoking-cessation medication [46]. Although black box warnings were issued by the FDA in 2009 for varenicline and bupropion regarding possible psychiatric effects and concern for cardiovascular safety, these were removed late in late 2016 [47, 48].

Our cohort may inherently be subject to poor documentation and treatment for tobacco use. The focus of most tobacco research has been in younger age cohorts even though the elderly are shown to benefit from tobacco cessation as evidenced by a reduction in risk of death from coronary heart disease, chronic obstructive lung disease and lung cancer [2, 32, 49, 50]. Additionally, the elderly are a growing population, have more frequent contact with the health care system than younger people and have the greatest potential for health care professionals to educate, recommend and influence to participate in smoking cessation programs. However, quit attempts decrease with increasing age [51, 52]. From 2005 to 2014, the decrease in tobacco use among persons ≥65 years was small (2.1%) compared to those aged 18–24 (46.6%) and 45–64 (22.6%) [4]. Also, by health insurance coverage, Medicare was the only group that did not decrease tobacco use from 2005 to 2014 [4]. This is in spite of evidence that older smokers are more likely than younger smokers to quit and maintain abstinence [49, 50].

Our study has several limitations. First, our cohort was limited to Medicare beneficiaries aged > 65 years with Medicare Parts A and B coverage and our findings may not be relevant to younger patients or individuals with Medicare enrolled in HMOs. A major limitation is that we cannot determine the true sensitivity or specificity of Medicare data for diagnostic coding of tobacco use. We compared rates generated from Medicare tobacco use diagnostic codes to rates generated by self-report via telephone survey (BRFSS), but the comparison is at the level of the group, not the individual. Self-reported cigarette smoking has been shown to correlate highly with serum cotinine levels and found to accurately identify smokers [42]. Medicare data probably also underestimates the rate of physician counselling for tobacco cessation. In one study, 71.0% of patients reported receiving cessation counselling but only 46.2% of physicians documented counselling (35).

## Conclusion

In summary, our study shows an increase in the tobacco use diagnoses among Medicare beneficiaries which coincided with greater efforts on multiple fronts to identify and intervene in tobacco dependence. Nevertheless, estimates of current tobacco use or history of tobacco use based on Medicare data substantially underestimate their real prevalence. Medicare data cannot be used to track prevalence of tobacco use

## Data Availability

The BRFSS data obtained for the study is publically available at their website. The data that support the CMS findings of this study are available from CMS but restrictions apply to the availability of these data, which were used under license for the current study, and so are not publicly available. Data are however available from the authors upon reasonable request and with permission of CMS.

## Abbreviations

BRFSS: *Behavioral Risk Factor Surveillance System*
CMS: Center for Medicare and Medicaid Services
HCPCS: Healthcare Common Procedure Coding System
ICD-9: International Classification of Disease, Ninth Revision
